# Association of Exercise-Based Cardiac Rehabilitation with Progression of Paroxysmal to Sustained Atrial Fibrillation

**DOI:** 10.3390/jcm10030435

**Published:** 2021-01-23

**Authors:** Benjamin J. R. Buckley, Stephanie L. Harrison, Elnara Fazio-Eynullayeva, Paula Underhill, Deirdre A. Lane, Dick H. J. Thijssen, Gregory Y. H. Lip

**Affiliations:** 1 Cardiovascular and Metabolic Medicine, Institute of Life Course and Medical Sciences, Faculty of Health & Life Sciences, William Henry Duncan Building, Liverpool Centre for Cardiovascular Science, University of Liverpool and Liverpool Heart and Chest Hospital, Liverpool L7 8TX, UK; Stephanie.Harrison@liverpool.ac.uk (S.L.H.); Deirdre.Lane@liverpool.ac.uk (D.A.L.); Gregory.Lip@liverpool.ac.uk (G.Y.H.L.); 2 TriNetX LLC., 25 Cambridge Park Dr #500, Cambridge, MA 02140, USA; Elnara.Fazio@trinetx.com; 3 TriNetX LLC., The Leadenhall Building Level 30, 122 Leadenhall Street, London EC3V 4AB, UK; Paula.Underhill@trinetx.com; 4 Aalborg Thrombosis Research Unit, Department of Clinical Medicine, Aalborg University, 9000 Aalborg, Denmark; 5 Liverpool Centre for Cardiovascular Science, Liverpool John Moores University, Liverpool L7 8TX, UK; D.Thijssen@ljmu.ac.uk; 6 Department of Physiology, Research Institute for Health Science, Radboud University Medical Centerum, 6525 GA Nijmegen, The Netherlands

**Keywords:** atrial fibrillation, cardiac rehabilitation, secondary prevention, disease progression, rehabilitation

## Abstract

Progression of atrial fibrillation (AF) is associated with worsened prognosis for cardiovascular events and mortality. Exercise-based-cardiac rehabilitation programmes have shown preliminary promise for primary and secondary prevention of AF. Yet, such interventions are typically reserved for patients with acute coronary syndrome or undergoing revascularization. Using a retrospective cohort design, the present study investigated the association of exercise-based cardiac rehabilitation on the progression of paroxysmal to sustained AF, compared to propensity-matched controls. Patients with a diagnosis of paroxysmal AF were compared between those with and without an electronic medical record of exercise-based cardiac rehabilitation within 6-months of diagnosis. Using cox regression models, we ascertained odds of 2-year incidence for AF progression. This cohort of 9808 patients with paroxysmal AF demonstrated that exercise-based cardiac rehabilitation was associated with 26% lower odds of AF progression (odds ratio 0.74, 95% CI 0.66–0.83) compared to propensity-matched controls. This beneficial effect seemed to vary across patient subgroups. In conclusion, findings revealed that exercise-based cardiac rehabilitation was associated with significantly lower odds of progression from paroxysmal to sustained AF at 2-years follow-up compared to propensity-matched controls.

## 1. Introduction

Atrial fibrillation (AF) can present as one of three main clinical subtypes: paroxysmal AF (PAF; episodes of arrhythmia that terminate spontaneously), persistent AF (episodes that continue for >7 days and are not self-terminating), and permanent AF (ongoing long-term episodes). AF typically progresses from PAF to sustained AF (SAF; persistent or permanent AF), with a worsened prognosis for death, heart failure, and stroke during the peri-progression period [1].

Although excessive levels of vigorous exercise may be associated with increased incident AF, guideline levels of physical activity and exercise training have been shown to elicit potent AF-specific protection [2]. Indeed, 1500 metabolic equivalents (METs)-mins/week were associated with reduced risk of incident AF in men and even exceeding these recommendations (5000 MET-mins/week) were AF-protective in females [3]. Further, exercise-based cardiac rehabilitation (CR), in association with optimized drug therapy, can improve sinus rhythm maintenance in patients with SAF [4]. However, little is known about the impact of CR and exercise programmes on disease progression in those with PAF. Therefore, we investigated the association between exercise-based CR and the progression of PAF to SAF, compared to matched PAF patients without exercise-based CR.

## 2. Materials and Methods

This retrospective observational study was conducted on October 29, 2020 with anonymized data provided by TriNetX, a global federated health research network with access to electronic medical records (EMR) from participating academic medical centres, specialty physician practices, and community hospitals, predominantly in the United States. AF was identified from International Classification of Diseases, Tenth Revision, Clinical Modification (ICD-10-CM) codes in patient EMRs: I48.0 (PAF), I48.1 and I48.21 (SAF). Restrictions on the search dates were used so that all included patients had an EMR of PAF recorded at-least 2-years ago (to allow for follow-up). Exercise-based CR was identified from ICD-10-CM codes Z71.82 (Exercise counselling), Healthcare Common Procedure Coding System (HCPCS) code S9472 (CR program, non-physician provider, per diem), or Current Procedural Terminology (CPT) code 1013171 (Physician or other qualified health care professional services for outpatient CR). Correspondingly, these exercise-based CR codes were excluded in the matched controls. The exercise-based CR cohort were aged ≥18 years with exercise-based CR recorded in EMRs within 6-months of PAF diagnosis. Controls were aged ≥18 years with PAF and no EMR history of exercise-based CR. At the time of the search, 39 participating healthcare organizations had data available for patients who met the study inclusion criteria.

Baseline characteristics were compared using chi-squared tests or independent-sample *t*-tests. Current CR provision is typically reserved for cardiovascular patients following an acute coronary syndrome, those undergoing a revascularization procedure (coronary artery bypass graft or planned percutaneous coronary intervention), and patients with heart failure. Thus, propensity score matching was used to control for these differences in the two cohorts. Using logistic regression, exercise-based CR patients were 1:1 propensity score-matched with controls for age at PAF diagnosis, sex, race, hypertensive diseases, ischaemic heart diseases, heart failure, cerebrovascular diseases, diabetes mellitus, chronic kidney disease, cardiovascular procedures (including cardiography, echocardiography, catheterization, cardiac devices, and electrophysiological procedures), and cardiovascular medications (including beta-blockers, antiarrhythmics, diuretics, antilipemic agents, antianginals, calcium channel blockers, and ACE inhibitors). Following propensity score matching, logistic regression produced odds ratios (OR) with 95% confidence intervals (CIs) for 2-year incidence of SAF (persistent/permanent AF), comparing exercise-based CR with non-CR controls. Additional sub-analyses (following propensity score matching) were conducted to produce ORs with 95% CIs to explore the effect of population subgroups (age, sex, and comorbidities) on the odds of AF progression between the exercise-based CR cohort and controls. Statistical significance was set at *p* < 0.05.

## 3. Results

In total, 379,966 patients with PAF from 39 US healthcare organizations met the inclusion criteria for the control group and 4905 patients with PAF met the inclusion criteria for the exercise-based CR cohort. Compared to controls, the exercise-based CR cohort were younger, had a lower proportion of females, had a higher proportion of White and Black or African American, and had a higher proportion of comorbidities (Table 1). Following propensity score matching, there were 4904 patients in each cohort (*n* = 9808 in total), which were overall, well balanced for age, race, sex, included comorbidities, cardiovascular procedures, and cardiovascular medications (Table 1).

Using the propensity score matched cohort, progression from PAF to SAF at 2-year follow-up from PAF diagnosis was proportionally lower with 19.3% (*n* = 617 of 3197 patients) in the exercise-based CR cohort compared to 24.5% (*n* = 909 of 3716 patients) in the matched controls (OR 0.74, 95% CI: 0.66–0.83).

Subgroup analyses comparing exercise-based CR with controls demonstrated lower odds of SAF progression in patients aged <75 years (OR 0.66, 95% CI: 0.56–0.78); male (OR 0.71, 95% CI: 0.62–0.83); patients without chronic kidney disease (OR 0.66, 95% CI: 0.56–0.79); patients with heart failure (OR 0.81, 95% CI: 0.71–0.93) and patients without heart failure (OR 0.72, 95% CI: 0.56–0.93); and patients without a history of stroke (OR 0.73, 95% CI: 0.64–0.83). Patients aged ≥75 years (OR 0.92, 95% CI: 0.77–1.09); female (0.97, 95% CI: 0.8–1.19); with chronic kidney disease (OR 0.96, 95% CI: 0.81–1.13); and with a history of stroke (OR 0.97, 95% CI: 0.73–1.29) did not demonstrate a significant association with reduced odds of SAF progression.

## 4. Limitations

A number of limitations are noteworthy. First, the data were collected from health care organization EMR databases and some co-morbidities may be underreported. Indeed, recording of ICD codes in administrative datasets may vary by factors such as age, number of comorbidities, severity of illness, length of hospitalization, and whether in-hospital death occurred. In particular, an EMR of exercise-based CR does not provide information as to whether a patient attended, the intervention type and dose, or intervention adherence. We could also not determine the influence of attending different healthcare organizations due to data privacy restrictions. Further, the current AF classification scheme supported by the American Heart Association (AHA), American College of Cardiology (ACC), and the European Society of Cardiology (ESC) relies on duration of rhythm and spontaneous conversion with patients considered to have paroxysmal, persistent, or permanent AF. However, the accuracy and reliability of this classification scheme, especially in EMRs, is unknown. Second, the data were from multiple healthcare organizations in the United States, which may not be representative of the wider population, thus the generalizability of the results beyond this cohort is unclear. Third, residual confounding may have impacted our results, including lifestyle factors and socioeconomic status, which were not available from EMRs. Fourth, it was not possible to factor for multiple comparisons in the subgroup analyses within the online database. Finally, subsequent randomized controlled trials are needed to further investigate the causal impact of exercise-based CR on AF-specific outcomes.

## 5. Conclusions

The present study of 9808 patients with PAF demonstrates that exercise-based CR was associated with 26% lower odds of progression to SAF at 2-years, compared to propensity score matched controls. Since progression from PAF to SAF is associated with a further increased risk in mortality and morbidity, our observation of delayed progression has clinical value. Despite the promise of exercise as a primary and secondary therapy for AF, CR is typically not prescribed for patients with AF. Our findings provide support for the inclusion of patients with AF in exercise-based CR programmes for AF-specific protection.

## Figures and Tables

**Table 1:** Baseline characteristics % (*n*) * of the AF populations with and without CR and exercise before and after propensity score matching.

	Initial Populations			Propensity Score Matched Populations		
	AF without CR (*n* = 379,966)	AF with CR (*n* = 4905)	*p*-Value	AF without CR (*n* = 4904)	AF with CR (*n* = 4904)	*p*-Value
Age (years) at diagnoses; mean (SD)	70.6 (12.5)	69.6 (11.2)	<0.001	69.7 (11)	69.7 (11.2)	0.766
Female	45.4 (172,581)	32.4 (1587)	<0.001	32.1 (1574)	32.4 (1587)	0.779
Race ^a^						
White	84.4 (320,653)	87.1 (4272)	<0.001	88.2 (4326)	87.1 (4270)	0.087
Black or African American	7.4 (28,052)	8.5 (416)	0.004	8.3 (408)	8.5 (416)	0.771
Asian	1.3 (4937)	1.5 (72)	0.303	1.1 (54)	1.5 (72)	0.107
Unknown	6.8 (25,731)	2.8 (138)	<0.001	2.4 (116)	2.8 (138)	0.162
Hypertensive diseases	46.8 (177,861)	83.4 (4090)	<0.001	83.4 (4089)	83.4 (4088)	0.978
Ischaemic heart diseases	23.9 (91,001)	77.4 (3795)	<0.001	77.7 (3811)	77.4 (3793)	0.664
Cerebrovascular diseases	10.1 (38,352)	24.1 (1180)	<0.001	24.1 (1183)	24 (1179)	0.925
Diabetes Mellitus	18 (68,336)	37.3 (1830)	<0.001	37.1 (1819)	37.3 (1830)	0.818
Chronic Kidney Disease	12.2 (46,533)	30.5 (1497)	<0.001	30.3 (1490)	30.5 (1495)	0.913
Cardiovascular Procedures ^b^	38.7 (147,131)	89.1 (4372)	<0.001	88.7 (4351)	89.1 (4370)	0.543
Catheter ablation	0.5 (1959)	1.6 (79)	<0.001	1.0 (51)	1.6 (79)	0.001
Cardiovascular Medications ^c^	57 (216,644)	93.5 (4588)	<0.001	93.5 (4584)	93.5 (4586)	0.935
Most common antiarrhythmic drugs						
Lidocaine	18.7 (70,923)	64.3 (3153)	<0.001	64.3 (3154)	64.3 (3151)	0.946
Amiodarone	8.4 (32,044)	45.4 (2226)	<0.001	45.6 (2237)	45.4 (2224)	0.786
Adenosine	1.0 (3755)	8.1 (396)	<0.001	6.4 (315)	8.0 (394)	0.002
Dofetilide	1.0 (3860)	<0.1 (95)	<0.001	<0.1 (79)	<0.1 (94)	0.250

* Values are % (*n*) unless otherwise stated. Baseline characteristics were compared using a chi-squared test for categorical variables and an independent-sample *t*-test for continuous variables. ^a^ Data are taken from structured fields in the electronic medical record systems of the participating healthcare organizations, therefore, there may be regional or country-specific differences in how race categories are defined. ^b^ Cardiovascular procedures include cardiography, echocardiography, catheterization, cardiac devices, electrophysiological procedures. ^c^ Cardiovascular medications include beta-blockers, antiarrhythmics, diuretics, antilipemic agents, antianginals, calcium channel blockers, Angiotensin-converting-enzyme (ACE) inhibitors. AF; atrial fibrillation, CR; cardiac rehabilitation and exercise programmes, SD; standard deviation.

## Data Availability

To gain access to the data in the TriNetX research network, a request can be made to TriNetX (https://live.trinetx.com), but costs may be incurred, a data sharing agreement would be necessary, and no patient identifiable information can be obtained.
